# 3D Da Vinci robotic surgery: is it a risk to the surgeon’s eye health?

**DOI:** 10.1007/s11701-023-01604-z

**Published:** 2023-04-27

**Authors:** Fernando Molle, Maria Cristina Savastano, Federico Giannuzzi, Claudia Fossataro, Davide Brando, Andrea Molle, Maria Teresa Rebecchi, Benedetto Falsini, Roberta Mattei, Giorgia Mirisola, Eleonora Poretti, Valentina Cestrone, Elena D’Agostino, Pierfrancesco Bassi, Giovanni Scambia, Stanislao Rizzo

**Affiliations:** 1 Ophthalmology Unit, “Fondazione Policlinico Universitario A. Gemelli, IRCCS”, Largo A. Gemelli, 8, 00168 Rome, Italy; 2 Catholic University “Sacro Cuore”, 00168 Rome, Italy; 3 Urology Department, Sacro Cuore Catholic University, A. Gemelli University Hospital, 00168 Rome, Italy; 4 Department of Woman, Child, and Public Health, Fondazione Policlinico Universitario A Gemelli IRCCS, 00168 Rome, Italy

**Keywords:** Stereopsis, 3D visual system, Surgical system, Robotic surgery

## Abstract

Da Vinci three-dimensional (3D) system has been increasingly used in customary surgical settings, gaining fundamental relevance for abdominal, urological, and gynecological laparoscopic surgery. The aim of this research is to evaluate the degree of discomfort and potential changes in the binocular vision and ocular motility of surgical operators, who employ 3D vision systems during Da Vinci robotic surgery. Twenty-four surgeons were enrolled in the study, including twelve who typically use the 3D Da Vinci system and twelve who routinely employ 2D system. Routine general ophthalmological and orthoptic examinations were conducted at baseline (T0), the day before surgery, and 30 min after the 3D or 2D surgery (T1). In addition, surgeons were interviewed using a questionnaire of 18 symptoms, with each item containing three questions regarding the frequency, severity, and bothersomeness of the symptoms, in order to evaluate the degree of discomfort. Mean age at evaluation was 45.28 ± 8.71 years (range 33–63 years). Cover test, uncover test, and fusional amplitude showed no statistically significant difference. After surgery, no statistical difference was observed in the Da Vinci group on the TNO stereotest (*p* > 0.9999). However, the difference in the 2D group resulted statistically significant (*p* = 0.0156). Comparing participants (*p* 0.0001) and time (T0–T1; *p* = 0.0137), the difference between the two groups was statistically significant. Surgeons using 2D systems reported more discomfort than those using 3D systems. The absence of short-term consequences following surgery with the Da Vinci 3D system is a promising conclusion, considering the numerous advantages of this technology. Nonetheless, multicenter investigations and more studies are required to verify and interpret our findings.

## Introduction

Visual fatigue is a complex condition, resulting from a multitude of elements associated with the act of seeing, and furthermore results are difficult to demonstrate empirically. Numerous quantitative and qualitative research on visual fatigue have been undertaken on people working with video display units (VDUs) [1].

In contrast to ocular and general pain, visual discomfort is usually caused by problem on the accommodative abilities and the necessary additional activity of the extraocular muscles [1]. In the last two decades, stereoscopic technologies have extremely spread in various sectors, such as the entertainment, medical, industrial, and scientific fields. In an effort to enhance depth perception and spatial orientation during operations, three-dimensional (3D) imaging systems have been developed. Nonetheless, some unfavorable consequences of stereoscopy on eye health have also been identified [2–4]. Frequently, the use of 3D imaging has been associated with headache and eye fatigue, sometimes being referred as visually induced motion sickness (VIMS) [5]. Experiencing 3D pictures may be possible, thanks to motor and sensory integration using regular binocular vision. Patients with abnormal binocular vision, such as those who suffer from strabismus, amblyopia, or anisometropia, may, nonetheless, show a wide range of fusional capacities [6].

The stimuli for accommodation and convergence are congruent in the usual setting, and when an item approaches the viewer, the stimuli for both activities increase. In contrast to the actual world, a 3D stereoscopical display creates a stimulus for convergence supplied by the distance to the geometric position of the picture rather than an accommodation stimulus produced by the distance of the image on the screen. A stereoscopic perception can only be produced when there is a mismatch between the vergence and accommodation stimuli, since they are always combined when the picture is situated geometrically on the same plane of the screen. This discrepancy has been cited as a key factor in the visual discomfort, referred experiencing 3D stereoscopic stimuli [7, 8].

The limited degrees of freedom and thus restricted mobility of the straight laparoscopic tools, the two-dimensional (2D) vision, and poor ergonomics for the surgeon are intrinsic issues to the current minimally invasive surgical procedures, resulting in a longer and harder learning curve. Thus, robotic systems have been developed to solve these issues [9].

Da Vinci (Intuitive Surgical, Mountain View, CA) consists of two parts. The surgeon manipulates instrument controls beneath a 3D binocular display, showing the operational field, while the joysticks control robotic arms on a three-armed patient side cart, imitating the 7-degrees-of-freedom human arm [10]. The Da Vinci robotic system overcomes hands trembling during manual surgery, and furthermore enables to perform routine minimally invasive surgical procedures more quickly and effectively than with conventional minimally invasive approaches [9, 11, 12].

In the recent years, the Da Vinci 3D system has been increasingly employed in the usual surgical settings, gaining fundamental relevance for abdominal, urological, and gynecological laparoscopic surgery. However, numerous surgeons using the 3D Da Vinci equipment reported post-operative ocular discomfort. Therefore, the aim of our prospective research was to evaluate ocular motility and binocular vision alterations in a cohort of various sub-specialties surgeons, before and after performing 3D surgery with Da Vinci robot assistance.

## Materials and methods

This is a single-center prospective research study, conducted at Fondazione Policlinico Universitario A. Gemelli, IRCCS, Rome, Italy. Two groups were identified. The former included surgeons using the 3D Da Vinci system, while the latter those using routine 2D surgical system. Surgeons were enrolled from the Urology Unit, Gynecology Oncology Unit, General Surgery Unit and Digestive Surgery Unit, who typically use the Da Vinci laparoscopic and robotic 3D vision system 1–3 times a week for at least 6 h/day for routine surgical procedures. The following exclusion criteria were applied: presence of anterior or posterior segment eye diseases, inducing permanent visual impairment, such as corneal diseases, cataracts, and maculopathies.

Twelve surgeons using routinely the Da Vinci 3D system were enrolled in the study. Twelve surgeons employing routinely the 2D operating microscope without any 3D visual device, were included in the study as controls.

Routine complete ophthalmological and orthoptic examinations including cover-uncover test, measuring eventual angle deviation, evaluation of ocular motility, fusional amplitude for near and far, fusional amplitude with and without Bagolini striated lenses, convergence, and stereopsis (TNO stereotest) were performed at baseline (T0), the day before surgery, and repeated 30 min after the 3D or 2D surgery in the same day (T1).

At the conclusion of the ophthalmic and orthoptic evaluations, a questionnaire was administered regarding the surgeons’ reported symptoms. The questionnaire, showed in Table 1, assesses 18 symptoms, with each item containing three questions about the frequency, severity, and bothersomeness of the symptoms, for a total of 54 items. For each query, the patient could select from three possible responses: never, sometimes, and often. The respective scores for these responses were 0, 1, and 2. The highest possible score was 108. Less than 54 was considered “no discomfort”, between 54 and 81, “minimal discomfort”, and between 81 and 108, “significant discomfort”.

**Table 1:** Questionnaire

No.	Symptoms	Frequency			Severity			Bothersomeness		
1	Light bothering	0	1	2	0	1	2	0	1	2
2	Foggy vision	0	1	2	0	1	2	0	1	2
3	Blurred vision	0	1	2	0	1	2	0	1	2
4	Double images	0	1	2	0	1	2	0	1	2
5	Haloes	0	1	2	0	1	2	0	1	2
6	Excessive blinking	0	1	2	0	1	2	0	1	2
7	Foreign body sensation	0	1	2	0	1	2	0	1	2
8	Lacrimation	0	1	2	0	1	2	0	1	2
9	Itchy eye	0	1	2	0	1	2	0	1	2
10	Burning eye	0	1	2	0	1	2	0	1	2
11	Red eye	0	1	2	0	1	2	0	1	2
12	Orbital pain	0	1	2	0	1	2	0	1	2
13	Ocular heaviness	0	1	2	0	1	2	0	1	2
14	Headache	0	1	2	0	1	2	0	1	2
15	Sickness	0	1	2	0	1	2	0	1	2
16	Fatigue	0	1	2	0	1	2	0	1	2
17	Dizziness	0	1	2	0	1	2	0	1	2
18	Difficulty in digesting	0	1	2	0	1	2	0	1	2

Clinical investigations were conducted in accordance with the ethical principles that have their origin in the Declaration of Helsinki (1964) and was approved by the Catholic University of the Sacred Heart's Ethical Committee in Rome, Italy (protocol code: 3735). Signed informed consent was obtained from each enrolled surgeon.

### Statistical analysis

The statistical analysis was conducted using GraphPad PRISM Software (Version 9.0; GraphPad, La Jolla, CA, USA). Our sample’s normality was determined using the Shapiro–Wilk test, and a *p* > 0.05 was used to confirm the null hypothesis. We performed a *t* test for unmatched pairs. To compare the difference between each pair of non-matched means, the Wilcoxon test, which computes confidence intervals, was used. To compare the difference between the two different groups between baseline and post-surgery parameters, we used the two-way ANOVA. For contingency analysis, chi-square and Fisher’s exact tests were performed. In addition, correlation studies were conducted on continuous variables. The quantitative results were represented as the mean ± standard deviation, and a *p* value < 0.05 was deemed statistically significant.

## Results

A total of 24 surgeons, 48 eyes, were analyzed. Seventeen were males (65.4%) and 9 females (34.6%). The mean age at evaluation was 48.25 ± 8.71 years (range 33–63 years). Average years of work as surgeon was 16.3. Best corrected visual acuity (BCVA), measured with Snellen visual acuity chart, was 20/20 in 48 eyes (100%). Seventeen (65.4%) wore eyeglasses or contact lenses (82.4% and 17.6% respectively). Demographic characteristics of surgeons are showed in Table 2.

**Table 2:** Demographic characteristics of surgeons

Variables	Population
Male; no. (%)	17 (65.4%)
Female; no. (%)	9 (34.6%)
Mean age at evaluation; years	48.25 ± 8.71 (range 33–63)
Average years of work	18.25 ± 8.43
Wearing eyeglasses or contact lenses; no. (%)	17 (65.4%)

**Fig. 1:**
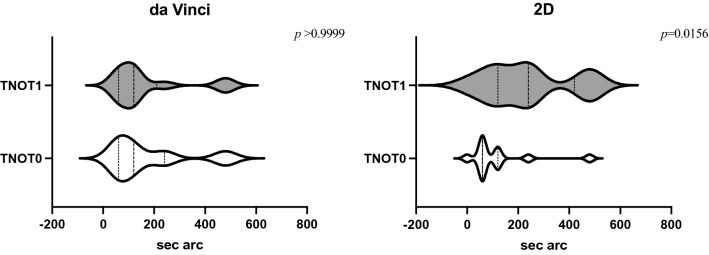
Violin plot showing the results at the baseline (T0) and at T1 for Da Vinci group (on the left) and “2D” group (on the right)

Fusional movements and convergence eye movements resulted normal and unchanged at T1. Mean value at the cover test performed at distance (6 m) was 0 Δ both at baseline and at T1 in both groups, whereas in Da Vinci group, mean value at the cover test performed at near was – 2.33 Δ at the baseline and – 2.83 Δ at T1. In the group using 2D system, mean value at the cover test performed at near was − 1.66 Δ at the baseline and – 1.83 Δ at T1. No statistical difference could be assessed at baseline for the cover test performed for far vision. Similarly, for the cover test performed for near vision in both the Da Vinci and 2D groups, none statistically significant difference was detected (*p* = 0.726 and *p* = 0.8125, respectively). Fusional amplitude performed in both the Da Vinci and 2D groups as well did not show any statistically significant difference (*p* = 0.4062 and *p* = 0.1035 for far vision, *p* = 0.7344 and *p* = 0.8301 for near vision, respectively). Likewise, for fusional amplitude performed with Bagolini striated lenses for far and near vision in both the Da Vinci and 2D groups, the difference was statistically non-significant (*p* = 0.4434 and *p* = 0.0938 for far vision, *p* = 0.7344 and p = 0.7930 for near vision, respectively). No statistical difference could be neither assessed after surgery at TNO stereotest in Da Vinci group (*p* > 0.9999), while the difference at TNO stereotest in 2D group was statistically significant (*p* = 0.0156), as it could be seen in Fig. 1.

**Fig. 2:**
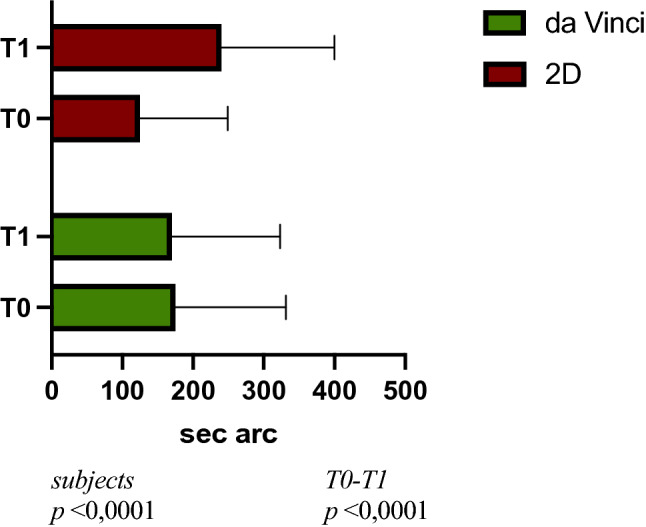
Bar plot showing the difference in TSO stereotest (sec arc) between the two groups at the baseline (T0) and T1

Moreover, two-way ANOVA was used to compare the difference between the two groups at T0, before surgery, and T1, after surgery. It emerged a statistically significant difference between the two groups comparing both subjects (*p* < 0.0001) and timing (T0-T1; *p* = 0.0137). Mean value at TNO stereotest was 172.5 s arc for Da Vinci group and 182.5 s arc for 2D group (difference between means was − 10.00 s arc). Results are shown in Fig. 2.

Concerning the administered questionnaire, 8 of twelve surgeons who had used the Da Vinci system had a score less than 54, indicating no discomfort, and 7 of those reported a score of less than 10 points, whereas 4 of the twelve surgeons reported a score between 54 and 81, indicating minimal discomfort. None of the patients who utilized the Da Vinci system reported a significant level of discomfort.

In contrast, among the surgeons who used the 2D system, 6 reported a score of less than 54, 4 reported a score between 54 and 81, showing minimal discomfort, and two reported a score higher than 81, indicating significant discomfort.

## Discussion

The primary purpose of the robotic surgery is to improve surgical processes, by increasing the surgeon’s vision and control during operations. An improved 3D vision, higher magnification of the surgical field, improved hand eye coordination, a largely bloodless field, removal of the surgeon’s hands tremors, decreased surgeon discomfort are all benefits deriving from robot-assisted surgery[13]. All of these elements contribute to a more accurate surgical dissection, thanks to better stereoscopic vision with depth perception, which is essential to improve intraoperative and post-operative outcomes [14]. As demonstrated by O’Connor et al. [15], stereoacuity plays a crucial role in high level motor skills acquisition. Thus, the ability to perform fine motor tasks is improved in a measurable way by the presence of stereopsis. As Kulp et al. reported [16], both adults, as the enrolled surgeons in our study, and pediatric population get advantages from stereopsis, performing daily life, working and scholar motor tasks [16, 17].

The relevance of stereopsis in being able to view a 3D picture has been supported by several research in the literature [6]. The inability of children with strabismus and amblyopia to observe a 3D picture was demonstrated by Nishina et al. [18], showing the necessity for laminar processes that allow the brain to rebuild the image at the cortex level. Cao et al. [19, 20] demonstrated that the analog qualities of 3D visual perceptions may be produced by spiking neurons, which interact in hierarchically organized laminar circuits of the visual cortex. Stereopsis is specifically influenced by interactions between layers 4 and 3B and layers 2 and 3A in V1 and V2, which also explains how binocular and monocular information combines to produce 3D boundary and surface perceptions [21]. Nevertheless, studies on the effect of the 3D system on visuoperceptive functions are still extremely limited in the literature, this is the reason why we would like to investigate this field. Howarth and coworkers [3] demonstrated that viewing stereoscopic 3D pictures can be more associated with asthenopic symptoms and visually induced motion sickness than watching 2D images [5, 6]. Tuna et al. [22] found that presbyopia and hypermetropia negatively affect surgeons' 3D vision; thus, they deeply recommend to use the proper refractive correction when performing robotic surgeries.

According to our results, the 3D robotic technology of the Da Vinci system seems to have no effect in the immediate post-operative period, i.e., 30 min after performed surgery, as evidenced by the unchanged stereopsis parameters, measured by the TNO stereotest, and the non-statistically significant changes in convergence phenomena, fusion movements, cover and uncover test and fusion amplitudes for distance and near, both with and without Bagolini striated lenses. In addition, although the sample size is small, it is important to point out that 7 of the surgeons who used the Da Vinci reported a score of less than 10, indicating a high level of instrument compliance.

However, as O'Connor referred, new stereopsis analysis methods are being developed. Among these, emerges the Asteroid test (accurate stereotest on a mobile device), which displays stimuli on an auto-stereoscopic 3D tablet while actively monitoring the test distance and adjusting disparity appropriately, with sub-pixel disparity levels shown using anti-aliasing [23, 24].

Of note, the effect of 2D system surgery on visual function was the most remarkable outcome of our study. As we demonstrated, indeed, the 2D surgery altered surgeons’ stereopsis in a statistically significant way.

These findings are also consistent with the results of the questionnaire, which revealed that the 2D system caused more distress than the 3D Da Vinci system. Nevertheless, to definitely confirm this important evidence a larger sample may be necessary.

Moreover, further studies could help us understand if stereoacuity may be connected with higher surgical expertise, whereas impaired stereoacuity may slow down surgical skills acquisition.

Additionally, as Biddle [25] demonstrated, different values of stereoacuity result using alternative tests. One potential limit of our study was that we did not perform other stereotests than the TNO to evaluate the stereoacuity. Few other limitations may be the lack of long-term follow-up, the small number of enrolled surgeons and different kind and duration of surgery performed which do not permit to draw definite conclusions.

## Conclusion

The absence of short-term effects after Da Vinci 3D system surgery can be evaluated as a good outcome, considering the many benefits of this technique, and, on the other hand, the stereopsis alterations caused by the two-dimensional visualization system. However, multicenter studies, in order to implement the sample, and further tests are needed to validate and deeply investigate our results.

## Data Availability

The data that support the findings of this study are available from the corresponding author, Federico Giannuzzi, upon reasonable request.
